# Serial lung and IVC ultrasound in the assessment of congestive heart failure

**DOI:** 10.1186/s13089-017-0062-3

**Published:** 2017-03-07

**Authors:** Rachel Spevack, Mohamed Al Shukairi, Dev Jayaraman, Jerrald Dankoff, Lawrence Rudski, Jed Lipes

**Affiliations:** 1 Internal Medicine Training Program, Department of Medicine, Jewish General Hospital, McGill University, 3755 Chemin de la Cote St Catherine, Montreal, H3T 1E2 Canada; 2 Critical Care Fellowship Training Program, McGill University, Montreal, Canada; 3 Division of General Internal Medicine, Department of Medicine, MUHC, McGill University & Adult Critical Care, Jewish General Hospital, McGill University, Montreal, Canada; 4 Department of Emergency Medicine, Jewish General Hospital, McGill University, Montreal, Canada; 5 Division of Cardiology, Department of Medicine, Jewish General Hospital, McGill University, Montreal, Canada; 6 Department of Adult Critical Care, Jewish General Hospital, McGill University, Montreal, Canada; 7 Division of General Internal Medicine, Department of Adult Critical Care, Jewish General Hospital, McGill University, Montreal, Canada

**Keywords:** Vascular ultrasound, Lung ultrasound, Inferior vena cava, B lines, Heart failure

## Abstract

**Background:**

Management of congestive heart failure (CHF) is dependent on clinical assessments of volume status, which are subjective and imprecise. Point-of-care ultrasound (POCUS) is useful in the diagnosis of CHF, but how POCUS findings correlate with therapy remains unknown. This study aimed to determine whether the changes in clinical evaluation of CHF with treatment are mirrored with changes in the number of B lines on lung ultrasound (LUS) and inferior vena cava (IVC) size. In this prospective observational study, investigators performed serial clinical and ultrasound assessments within 24 h of admission (T1), day 1 in hospital (T2) and within 24 h of discharge (T3). Clinical assessments included an evaluation of the jugular venous distension (JVD), hepatojugular reflux (HJR), pulmonary rales and a clinical congestion score was calculated. Ultrasound assessment included the IVC size and collapsibility, and the number of B lines in an 8-point scan.

**Results:**

Fifty consecutive patients were recruited with a mean age of 71.2 years (SD 12.7). Mean clinical congestion score on admission was 5.6 (SD 1.4) and declined significantly over time to 1.3 (0.91), as did the JVP, HJR and pulmonary rales. No significant changes were found in the IVC size between T1 [1.9 (0.65)] and T3 [2.0 (0.50)] or in the IVC collapsibility index [T1 0.3 (0.19) versus T3 0.25 (0.16)]. The mean number of B lines decreased from 11 (6.1) at T1 to 8.3 (5.5) at T3, although this decrease did not reach statistical significance. Spearman correlation between JVP and HJR versus IVC collapsibility and total B lines did not yield significant results.

**Conclusions:**

Clinical exam findings correlate over time during the management of CHF, whereas LUS and IVC results did not. The number of B lines did decrease with therapy, but did not reach statistical significance likely because the sampled population was small and had only mild heart failure. Further studies are warranted to further explore the use of lung ultrasound in this patient population.

## Background

Cardiovascular disease is the leading cause of mortality, representing 29% of all deaths, and costs the healthcare system 20.9 billion dollars annually in North America [1]. Congestive heart failure (CHF) is a complex clinical syndrome characterized by recurrent episodes of acute decompensation, requiring frequent hospitalizations and readmissions [2]. The diagnosis and management of CHF remain a clinical challenge; decisions are based on clinical assessments of volume status, which are used to estimate cardiac filling pressures [3]. Chest X-ray (CXR) has traditionally been used to suggest pulmonary congestion, but assessments can be subjective [4] and lack sensitivity and specificity [5]. Recently, serial assessments of brain natriuretic peptide (BNP) have been shown to correlate with the degree of hemodynamic congestion [6] and may be a reliable tool to guide management in heart failure patients [7].

The gold standard for determining cardiac congestion is cardiac catheterization with determination of right atrial and left atrial pressures, as estimated by the pulmonary capillary wedge pressures. These tests are invasive, time consuming and carry risk, and are not typically performed in patients admitted with decompensated heart failure [8]. As a result, physicians rely on clinical assessments to diagnose elevated cardiac filling pressures. Clinical evaluation, however, correlates poorly with invasive hemodynamics [9] and the physical exam lacks sensitivity and specificity for the diagnosis of heart failure, limiting its clinical utility [10].

Point of care ultrasound (POCUS) has been developed as an easy-to-learn, rapid, portable, and non-invasive adjunct to physical examination. Previous studies have shown that inferior vena cava size correlates with right atrial pressure and pulmonary capillary wedge pressure with good sensitivity and specificity [11, 12], and outperforms the physical exam in the detection of an elevated jugular venous pressure [13]. However, IVC as a surrogate for volume status has been increasingly questioned, as the correlation to CVP has been variable [14]. However, the American Society of Echo guidelines still recommend its use as a non-invasive measurement of CVP [15].

Similarly, lung ultrasound and B lines, an artifact representing extravascular lung water, similarly correlate significantly with pulmonary artery systolic pressure [16] and outperform classical signs and symptoms of congestion [17]. Both lung [18, 19] and vascular ultrasound [20–22] have been shown to be helpful in the assessment of undifferentiated dyspnea and in the diagnosis of heart failure. It is not clear how POCUS findings change with treatment.

## Methods

The primary objective of the current study is to compare serial lung and vascular ultrasound exam with clinical findings upon CHF treatment. The secondary objective was to explore the relationship between the severity of ultrasound-measured degree of congestion and length of hospital stay, hospital readmission and mortality.

**Fig. 1:**
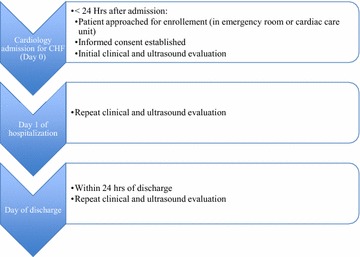
Study design with three separate time points for ultrasound and clinical examination

The current study recruited consecutive patients with a clinical diagnosis of left-sided congestive heart failure who require admission to the cardiology service, the admitting cardiology team having made the diagnosis. All hospitalized, spontaneously breathing adult patients, between the ages of 18 and 90, with an admitting diagnosis of either systolic or diastolic left-sided heart failure as per the admitting cardiologist were included. Exclusion criteria were as follows: patients undergoing invasive ventilation, patients with a primary diagnosis of right-sided heart failure, patients with active lung cancer, pulmonary fibrosis, interstitial lung disease, or pneumonia, patients who we are unable to have an ultrasound within the first 24 h of admission and patients who are not competent to sign the consent form. Patients underwent serial clinical and ultrasound assessments at three time points: within 24 h of admission (T1), day 1 of hospitalization (T2), and within 24 h prior to discharge (T3), for a total of three separate clinical and ultrasound exams (Fig. 1). The clinical assessments including the physical exam were performed by a senior resident in internal medicine and a senior critical care fellow. The assessors were aware of the time point of assessment.

Baseline patient demographics, including age, weight, sex, medical comorbidities, and etiology of heart failure were recorded from chart review. Laboratory values including hemoglobin, renal function and troponin values on admission were recorded from a chart review. Left ventricular ejection fraction (LVEF), diastolic function and valvulopathy were obtained from the most recent echocardiogram or during the current hospitalization. Chest X-ray (CXR) on admission was reviewed and graded by resident authors after they performed the clinical and ultrasound assessment as having interstitial edema or alveolar edema, where alveolar edema indicates more significant pulmonary congestion. Three months following discharge, a chart review was performed to assess for length of hospital stay, hospital readmissions and 90-day mortality. The hospital ethics committee approved the current study.

### Clinical assessment

The Killip and clinical congestion score (CCS) were calculated for each patient at across the three time points. The Killip score (0–4) [23] was calculated to grade the severity of heart failure, with 0 indicating no heart failure and 4 indicating cardiogenic shock. Although this score has been designed specifically in the setting of acute myocardial infarction, it was used in this study as it is a widely known descriptor of the severity of heart failure.

The clinical congestion score (CCS) [24] was calculated based on pulmonary rales (scored 0–4), central venous pressure elevation (scored 0–4), S3 (presence 1, absence 0) and orthopnea (scored 0–4). The CCS has a maximum score of 13, where higher scores indicate more severe heart failure. The CCS was validated on an outpatient population with stable heart failure, and has been shown to correlate with echocardiographic findings of increased filling pressures [19].

Specifically, the following symptoms and physical exam findings were graded for the above scores as follows: the presence of dyspnea and PND was graded as present (1) or absent (0). Orthopnea was graded between 0 and 4, with a score of 0 indicating the need of one pillow during sleep, and 4 indicating at least one night spent sleeping in a sitting position. Central venous pressure elevation was graded as 0 if the crests of neither the internal nor the external jugular vein are visible above the clavicle, and 4 if the crests are visible at the earlobe with the patient assessed at 30–45°. The hepatojugular reflux was recorded as present (1) or absent (0). Rales/crackles were graded as being absent (0), present in less than 50% of the lung field (1), or more than 50% of the lung field (2). Peripheral edema was graded between 0 and 4 according to the depth of indentation at the ankle.

### Ultrasound assessment

The POCUS ultrasound assessment was performed with the Zonare zone system (Zonare Medical Systems Inc, Mountain View, Ca, USA). Two authors (RS and MS) collected the ultrasound data after a 3-h training session and 10 supervised scans with an intensive care specialist with advanced cardiac echo and formal training in lung ultrasound prior to enrollment of patients. Brennan et al. [25] have shown that internal medicine residents with minimal training, including 4 h of formal didactic ultrasound teaching, and 20 sonographer supervised acquisitions were able to obtain adequate images of the IVC in 90% of patients. Each patient underwent serial scans at the time of the clinical assessments. Two authors (MS and JL) blinded to clinical information analyzed the stored images. A priori it was established that findings on vascular and LUS were not disclosed to the treating team unless they found a significant unexpected life-threatening diagnoses, such as venous thrombus, complex pleural effusion suggestive of empyema, pneumothorax or cardiac tamponade. As a result, no treatment decisions regarding the patient’s heart failure were made based on the ultrasound findings.

The IVC was analyzed in the long axis via the subcostal window using a 4-MHz phased-array ultrasound transducer. The diameter of the intra-hepatic IVC within 1–2 cm of the right atrium and the response of the IVC to an inspiratory maneuvre was measured and recorded. The size of the IVC on expiration was recorded, and the IVC collapsibility index was calculated as (IVC size on expiration − IVC size on inspiration)/IVC size on expiration.

All lung ultrasounds were performed using a 6-MHz curved array probe and a 3-s clip was recorded in each quadrant. A bilateral 8-point LUS examination was performed to assess for B lines following the International consensus guidelines [8]. An oblique intercostal view was obtained to maximize the number of B lines, and the actual count was made at the moment the most B lines were visualized.

### Data analysis

Clinical and ultrasound variables were analyzed using a paired *t* test. Pearson correlations were used to compare clinical and ultrasound variables. The impact of ultrasound and clinical assessments on hospital LOS and 90-day mortality was evaluated using univariate and multivariate analyses.

## Results

Fifty patients were recruited in the study. Five patients were excluded because they had a primary diagnosis of pneumonia complicated by pulmonary edema, and one patient was excluded because CHF was not the final diagnosis. Mean age was 77 (SD 12.7); 52% were males. The mean LVEF was 47.8%, and mean highly sensitive troponin T (Roche) value on admission was 293 ng/L (SD 824). The etiology of CHF was ischemic (33%), followed by valvular (26%) and hypertensive (6%), whereas 22% had a new diagnosis of CHF when they were recruited (Table 1). Median hospital length of stay was 7 days (IQR 5–13), 24% of patients were readmitted within 30 days of discharge and there was a 12% mortality rate at 90 days. Seventy-two percent of patients completed the three scans.

**Table 1:** Baseline clinical characteristics at time of recruitment

	Mean age	Sex (% male)	LVEF (%)	Diastolic dysfunction (%)	CKD (%)	Hs-Tnt (ng/L)	Interstitial edema CXR (%)	Pulmonary edema CXR (%)	Creatinine (μmol/L)
Mean or %	77.2	52.3%	47	32.6	41	293	66	48	150
SD	12.7	–	19	–	–	824	–	–	115

**Fig. 2:**
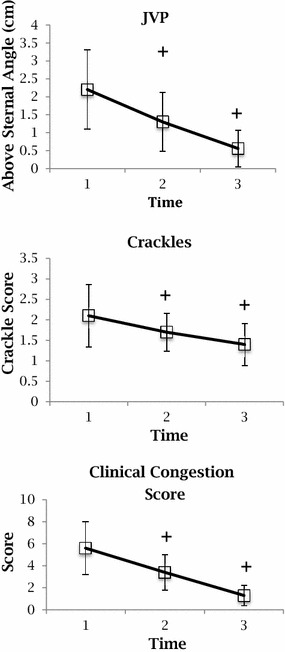
Clinical exam findings at each time point

The mean clinical congestion score on admission was 5.6 (SD 1.4) and decreased significantly over time to 1.3 (0.91) at T3 (Fig. 2). Other markers of clinical congestion, including orthopnea, dyspnea, JVD, hepatojugular reflux and pulmonary rales, also showed similar significant changes between T1 and T3. The median Killip score was 2.

**Fig. 3:**
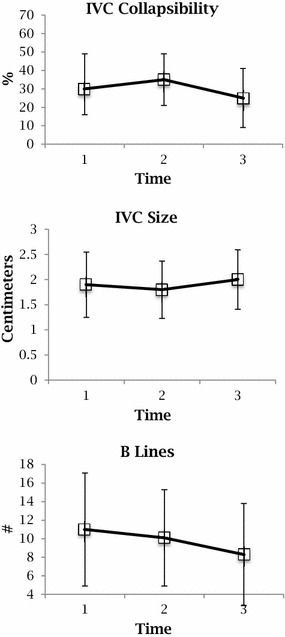
Ultrasound findings at each time point

The mean IVC size on expiration showed no significant change between T1 (1.9: SD 0.65) and T3 (2: SD 0.50), nor did the IVC collapsibility index [T1 0.3 (0.19) versus T3 0.25 (0.16)] (Fig. 3). The mean number of B lines did decrease [T1 11 (6.1) versus T3 8.3 (5.5)], but this decrease did not reach statistical significance (Fig. 2). Analysis using non-parametric test for trends or ANOVA did not alter these conclusions.

Pearson correlations between JVP and HJR showed significant correlations at T1 (0.58, *p* < 0.0001) and T2 (0.55, *p* = 0.0003). However, correlations between JVP or HJR and IVC size or collapsibility did not reach significance. Similarly, pulmonary rales and number of B lines across all time points did not show significant correlations. Univariate and multivariate analysis did not show any clinical or ultrasound variables that correlate with hospital length of stay and 90-day mortality rate.

Ninety-eight percent of the IVC scans were interpretable and read; 1 out of 50 could not be read because of poor image quality. All LUS scans were interpretable and read offline. The quality of the scans was determined by expert opinion, an Intensive care specialist with advanced cardiac echo and formal training in lung ultrasound. There were no significant differences in the ultrasound variables between the two authors. Twenty-eight percent of patients did not complete all three time points.

## Discussion

The present study showed no significant change in IVC size or collapsibility index despite significant improvement in clinical signs of congestion in patients treated for acute decompensated heart failure. The results did show a trend toward significance for number of B lines; there was a 38% decrease in the number of B lines with the treatment of heart failure.

There are several explanations for the current data. This study enrolled patients upon admission to the cardiac care unit, which occurred between hours and days after their presentation to the emergency department. Consequently, treatment for heart failure was already initiated in the emergency department prior to our first ultrasound assessment. Unfortunately, our hospital database does not track delay to admission for these patients in particular. With this delay, the degree of hemodynamic congestion was likely diminished, and any appreciable changes in IVC size and B lines subsequent to the initial findings were too small to detect. This may indicate that IVC size and B lines clear rapidly with treatment and the sensitivity of this technique to detect such changes is on the level of hours in the acute setting. Additionally, the patients in the current study had a median Killip score of 2, which indicates that this patient population had only mild heart failure.

In contrast to our results, Goonewardena et al. [26] demonstrated significant reduction in IVC size and increases in collapsibility index with heart failure treatment. This study found larger IVCs at admission (2.3 versus 1.9 cm in the current study). However, IVC size and collapsibility indices at discharge were identical between the two studies (2 cm in both studies). This may suggest that the greatest change in IVC size occurs early following initial therapy, and likely why the current study did not reach significance for this primary endpoint. However, IVC size as a surrogate for volume status has increasingly been questioned. Another explanation for the current data is that IVC size may not accurately represent hypervolemic states, and thus may not be useful in the management of decompensated heart failure.

Volpicelli et al. [27] conducted a study where number of B lines was calculated at admission and discharge, and found significant clearing of B lines after treatment. However, this population had more severe heart failure than the current study. This study used an arbitrary clinical scale out of ten points to grade the clinical severity of heart failure which included the presence (1) or absence (0) of lower extremity edema, pulmonary rales and wheezing, jugular venous distension, orthopnea, high respiratory rates (>25 breaths/min) and low pulse oximeter saturation; median score was 8/10 on admission whereas in the current study, the mean CCS was 5.6 out of 13. In another study, Gargani et al. [27] showed a significant reduction in B lines with treatment from 48 on admission to 20 at discharge, compared to the current study which showed a non-significant reduction of B lines from 11 to 8.3. Both these studies found a larger number of B lines on admission and sampled patients with more severe heart failure. This may indicate that LUS is a useful indicator of improvement in pulmonary congestion in patients with severe heart failure, but may fail to identify significant changes in pulmonary congestion in patients with milder CHF.

Of note, Volpicelli [28] analyzed the B lines using a different method. Eleven regions were scanned, compared to eight regions in the current study. Additionally, a positive region was defined as number of B lines over 3, and they analyzed the data based on the number of positive regions as opposed to absolute number of B lines. However, we did not find a significant change even when our data were analyzed in a similar manner. There was no prospective strategy in the study protocol to quantify confluent B lines, and as a result, they were counted as a single B line. Although there is a suggested method to quantify confluent B lines by calculating the proportion of visualized pleural line/length of the confluent B lines [29], we felt that there is insufficient evidence to apply this technique at the current time. It is possible that we underestimated the overall quantity of B lines using this approach; however, this method was consistently used across all time points and should not affect the overall results.

There are several limitations in the current study. This was a small study with only 50 patients enrolled at a single center. We did not formally estimate sample size as there were no reliable data to estimate effect size in this population. However, based on our results, a sample size of approximately 150 would be required to detect a statistically significant difference (*α* 0.05 and *β* 0.20) for B lines and a sample size of approximately 1000 would be required for change in IVC diameter.

Additionally, there was a high dropout rate with 28% of patients not completing all three time points. The clinical assessments were not blinded; resident authors performing these assessments knew at which time point each patient was being assessed, which could have introduced bias.

As mentioned above, since treatment was initiated early upon presentation and prior to recruitment, the ability to detect a potential decrease in IVC size and number of B lines may have be reduced. The clinical score would be less influenced as resolution of peripheral edema and crackles would lag, and the clinical history of orthopnea and PND is retrospective.

Assessment of volume status is challenging and point of care ultrasound may provide greater sensitivity to detect subclinical congestion before overt clinical congestion occurs. While this may be true for patients with more severe heart failure, the current study suggests that lung and vascular ultrasound in patients with less severe heart failure may not be sensitive enough to detect such small changes in hemodynamic congestion. As a result, serial POCUS may perform less well in patients with mild versus more severe heart failure. Based on the current results, IVC size or collapsibility index is not an accurate marker of improvement in hemodynamic congestion. B lines may be a better marker of congestion as these did decrease with the treatment of heart failure as expected. The lack of statistical significance for the number of B lines may be a result of a small sample size, and a sampled population with less severe heart failure than previously published, and an initial small number of B lines. Another interpretation of our data is that given the significant correlation over time with clinical scores and not with ultrasound, it may suggest that clinical exam is more sensitive than ultrasound at detecting congestive symptoms, although this is hypothesis generating given the small sample size.

The current study is the only study to the authors’ knowledge, which directly compared lung and IVC ultrasound in the dynamic clinical setting of heart failure management after hospital admission. Further studies are warranted to better understand the utility of bedside ultrasound in patients with mild heart failure, and which ultrasound modality provides the most accurate assessment of hemodynamic congestion for both diagnostic and prognostic purposes.

## Conclusions

In conclusion, this study shows that vascular and lung ultrasound does not show significant changes with the management of heart failure in a hospitalized population with acute decompensated heart failure, although LUS shows a trend toward significance. This may indicate that vascular ultrasound parameters are less robust in patients with mild congestion and may show the greatest change during initial management.

Further larger scale studies are needed to explore how POCUS may be useful in the acute and sub-acute management of heart failure.

## Abbreviations

CHF: congestive heart failure
POCUS: point-of-care ultrasound
LUS: lung ultrasound
IVC: inferior vena cava
JVP: jugular venous pressure
HJR: hepatojugular reflux
CCS: clinical congestion score
